# Haematological values in homozygous sickle cell disease in steady state and haemoglobin phenotypes AA controls in Lagos, Nigeria

**DOI:** 10.1186/1756-0500-5-396

**Published:** 2012-08-01

**Authors:** Akinsegun Akinbami, Adedoyin Dosunmu, Adewumi Adediran, Olajumoke Oshinaike, Phillip Adebola, Olanrewaju Arogundade

**Affiliations:** 1 Department of Haematology and Blood Transfusion, Lagos State University, College of Medicine, Lagos, Nigeria; 2 Department of Medicine, Lagos State University, College of Medicine, Lagos, Nigeria; 3 Department of Haematology and Blood Transfusion, Faculty of Clinical Sciences, College of Medicine, University of Lagos, Lagos, Nigeria

**Keywords:** Haematological values, Homozygous sickle cell disease, Steady state, Haemoglobin phenotype AA

## Abstract

**Background:**

Sickle cell disease is a genetic abnormality involving the haemoglobin. Although, it is primarily a red cell disorders, the white blood cells and platelets are also affected by the mutation. The consequent haemoglobin S causes polymerization of haemoglobin resulting in haemolysis and anaemia. This study aims to provide baseline haematological values in sickle cell disease patients in steady state and compare the deviation from haemoglobin phenotype AA control values.

**Methods:**

A case–control study was conducted amongst homozygous sickle cell patients attending the sickle cell clinics of Lagos State University Teaching Hospital Ikeja and haemoglobin phenotype AA controls. About 4.5mls of blood sample was collected from each participant for full blood count analysis. All blood samples were screened for HIV and haemoglobin phenotypes confirmed using cellulose acetate haemoglobin electrophoresis at pH 8.6.

**Results:**

A total of 103 cases and 98 controls were enrolled. The overall mean haemoglobin concentration for cases was 7.93 ± 1.47 g/dl, packed cell volume 24.44 ± 4.68%, mean cell volume 81.52 ± 7.89 fl, and mean cell haemoglobin 26.50 ± 3.20 pg. While for controls, mean haemoglobin concentration was 13.83 ± 1.32 g/dl, packed cell volume 43.07 ± 3.95%, mean cell volume 86.90 ± 4.69 fl, and mean cell haemoglobin 28.50 ± 1.34 pg. The overall mean white blood cell counts for the cases was 10.27 ± 3.94 *10^3^/μl and platelet counts of 412.71 ± 145.09*10^3^/μl. While white blood cell count for the controls was 5.67 ± 1.59*10^3^/μl and platelet counts of 222.82 ± 57.62*10^3^/μl.

**Conclusion:**

Homozygous sickle cell disease patients have lower values of red cell parameters, but higher values of white cell and platelets counts compared to haemoglobin phenotype AA controls.

## Background

Sickle cell disease is a genetic abnormality involving the haemoglobin. Patients present with a wide spectrum of disorders because of a single-point mutation in which thymine substitute for adenine, thereby encoding valine instead of glutamine in the sixth position of the beta-chain. Haemoglobin S resulting from the substitution causes polymerization of haemoglobin and red cell sickling on exposure to low oxygen tension and unsickle on oxygenation.

The repeated sickling and unsickling damages the red cell membrane leading to irreversibly sickled red cell even when the oxygen pressure is increased thus reducing red cell life span as a result of membrane damage inducing anaemia. The white blood cells and platelets are also affected by the mutation.

### Red blood cells in sickle cell disease

Quantitative and qualitative changes in red blood cells have been reported. Haemolysis consequent to the damaged red cell membrane could be intravascular or extravascular. The former results from the lysis of complement-sensitive red cells [1] and haemoglobin lost during sickling-induced membrane damaged. [2,3] The latter, occurs by phagocytosis of red cells that have undergone sickling [4,5] and physical entrapment of rheologically compromised red cells.[6] Increased susceptibility to mechanically induced cell fragmentation has been documented in-vitro and in sickle cell patients undergoing vigorous exercise [3].

Degree of haemolysis is inversely related to haemoglobin concentration and packed cell volume in sickle cell anemia patient [7]. Numerous factors affect haemolysis in sickle cell anaemia, percentage of irreversible sickle cell is of greatest significance. [7] The degree of haemoglobin polymer formation, calculated from the mean corpuscular haemoglobin concentration and the relative proportion of haemoglobin fractions also correlates closely with the severity of haemolysis. [8,9].

### White blood cells in sickle cell disease

Although sickle cell disease is primarily a disease of the red blood cell, leucocytes, because of their sizes obstruct blood vessels more effectively than red blood cells when attached to the endothelium. The red blood cells measures 7.2 μm, while small lymphocytes measures 10 μm, neutrophils 10–14 μm , large lymphocytes 12–16 μm, monocytes 14-20 μm. Bacterial infection associated with leucocytosis is a known predisposing factor to sickle cell disease crises [10,11]. A high absolute neutrophil count showed statistically significant relationship with clinical severity of sickle cell anaemia [12].

Many complications of sickle cell disease are associated with leucocytosis. It is a risk factor for early sickle cell disease –related death. [13] It is implicated in clinically overt stroke [14,15].

Pathogenesis of silent cerebral infarction [16] and acute chest syndrome [17] have been associated with leucocytosis.

### Platelets in sickle cell disease

Unlike the red and white blood cells, the clinical effects of platelets on sickle cell disease are not well established [18]. However, an association between stroke in sickle cell disease and platelet count > 450,000/μl has been reported [19]. Qualitatively, poor platelet aggregation responses to epinephrine and ADP were also reported in sickle cell disease [20].

The objective of this study was to provide baseline haematological values in sickle cell anaemia patients in steady state and compare deviation of these parameters from normal controls with haemoglobin phenotypes AA.

## Methods

A case–control study was conducted amongst Sickle cell patients in steady state attending the sickle cell clinics of Lagos State University Teaching Hospital Ikeja (LASUTH) referred from private clinics outside the hospital and other clinics in the hospital and whose diagnoses were confirmed by alkaline haemoglobin electrophoresis and controls consisting of medical students, doctors and nurses of the institution between September to December 2011 after obtaining approval from LASUTH, the institution’s Ethics and Research Committee. Written and verbal consents were obtained from each participant.

### Study design

Sickle cell patients filled validated structured questionnaires including demographic information, history of previous blood transfusion, and surgery, previous history of crises, date of last crises, cigarettes and alcohol intake. Inclusion criteria were; patients with haemoglobin phenotype SS, no history of crises in the past 3 months established by a careful history and complete physical examination, no previous history of surgery, no history of blood transfusion in the past 3 months. Exclusion criteria were; history of blood transfusion in the past 3 months, haemoglobin phenotype SC patients, previous history of surgery, and HIV infected patients.

All controls were phenotype AA as confirmed by Hb electrophoresis and were asked to fill questionnaires containing demographic information. Controls with haemoglobin phenotype AS or SC were excluded from the study. All patients were on routine tablets used in the sickle cell clinics i.e. folic acid ,paludrine and vitamin B complex tablets, none of them was on hydroxyurea therapy which could impact on the reported results for the patient group.

### Collection of samples

A blood sample of 4.5mls was collected from both patients and controls into Ethylene Diamene Tetraacetic acid (EDTA) anticoagulant bottle for full blood count analysis done on the same day of collection using Sysmex KN-21 N,(manufactured by Sysmex corporation Kobe, Japan) a three- part auto analyzer able to run 19 parameters per sample including haemoglobin concentration, packed cell volume, red blood cell concentration, mean corpuscular haemoglobin, mean cell volume, mean corpuscular haemoglobin concentration, white blood cells and platelet parameters.

### Procedure

Well mixed blood sample was aspirated, by letting the equipment sampling probe into the blood sample and then pressing the start button. Approx. 20ul of blood was aspirated by the auto analyzer. Result of analysis is displayed after about 30secs.A printout copy of result is released on the thermal printing paper.

All blood samples were also screened for HIV using determine rapid kit [21], and haemoglobin phenotypes of all patients and controls were confirmed using cellulose acetate haemoglobin electrophoresis at pH 8.6. About 50 ul of EDTA blood samples of patients and controls were lysed with equal volume of water and haemoglobin separated out with carbon tetrachloride. Lysed samples of patients and controls and known AA, AS, SC, and SS controls were placed on cellulose acetate paper in batches using an applicator . The paper was placed in electrophoretic tank containing tris-buffer, pH 8.6,electric current was applied. All samples and AA, AS, SC, and SS controls samples migrated from negative to positive pole at 400volt. Haemoglobin separation occurs within 4-6minutes, haemoglobin bands were compared with the known controls.

### Statistical analysis

Data were analyzed using SPSS version 16.0 (Statistical Package for Social Sciences, Inc., Chicago, Ill). The descriptive data were given as means ± standard deviation (SD. The Pearson chi squared test was used for analytic assessment and the differences were considered to be statistically significant when the p value obtained was < 0.05.

## Results

### Demographic characteristics of cases and controls

A total of 103 cases and 98 controls were enrolled. Cases consisted of 57 (55.33%) females and 46 (44.66%) males, while controls were made up of 68 (69.38%) females and 30 (30.61%) males (Table 1).

**Table 1:** Gender, age and background information of participants

**Parameters**	**Cases**		**Controls**	
	Males	Females	Males	Females
Frequency	46(44.66%)	57(55.33%)	30(30.61%)	68(69.38%)
Mean age ± SD	22.62 ± 6.93	24.72 ± 8.37	28.74 ± 9.47	31.18 ± 10.49
Overall Mean age ± SD	23.79 ± 7.81	30.43 ± 10.19		
	Frequency	Percent		
Bone pain crises				
Yes	94	91.2		
No	9	8.73		
Total	103	100		
Cigarette smoking				
Yes	1	0.97		
No	102	99.03		
Total	103	100		
Alcohol intake				
Yes	11	10.67		
No	92	89.32		
Total	103	100		

The overall mean age of the cases was 23.79 ± 7.81 years, and control was 30.43 ± 10.19 years. The minimum age of cases was 14 and maximum of 44 years and for controls were 17 years and 57 years respectively. All participants tested negative to HIV antibodies.

### Hematological indices in study population

For cases, the overall mean haemoglobin concentration was 7.93 ± 1.47 g/dl, packed cell volume 24.44 ± 4.68%, mean cell volume 81.52 ± 7.89 fl, mean cell haemoglobin 26.50 ± 3.20 pg, and mean cell haemoglobin concentration 32.50 ± 1.07 g/dl. While for controls, mean haemoglobin concentration was 13.83 ± 1.32 g/dl, packed cell volume 43.07 ± 3.95%, mean cell volume 86.90 ± 4.69 fl, mean cell haemoglobin 28.50 ± 1.34 pg, and mean cell haemoglobin concentration 32.06 ± 0.90 g/dl (Table 2).

**Table 2:** Mean values of full blood count parameters

**Parameters**	**Cases**			**Controls**		
	**Males**	**Females**	**Overall**	**Males**	**Females**	**Overall**
Hgb g/dl	8.11 ± 1.53	7.78 ± 1.42	7.93 ± 1.4	13.83 ± 1.32	11.96 ± 3.10	13.83 ± 1.32
PCV%	25.04 ± 4.90	23.95 ± 4.49	24.44 ± 4.68	43.07 ± 3.95	36.59 ± 3.30	43.07 ± 3.95
MCV fl	81.71 ± 6.34	81.36 ± 9.01	81.52 ± 7.89	86.90 ± 4.69	84.63 ± 8.82	86.90 ± 4.69
MCH pg	26.54 ± 2.46	26.47 ± 3.71	26.50 ± 3.20	28.25 ± 1.34	27.20 ± 1.92	28.50 ± 1.34
MCHCg/dl	32.45 ± 1.00	32.54 ± 1.08	32.52 ± 1.07	32.06 ± 0.90	31.59 ± 0.93	32.06 ± 0.90
WBC	10.82 ± 4.95	9.83 ± 2.86	10.27 ± 3.94	5.75 ± 1.63	5.63 ± 1.59	5.67 ± 1.59
Plts	408.40 ± 133.42	416.21 ± 155.09	412.71 ± 145	239 ± 62.25	222.15 ± 58.03	222.82 ± 57

Abbreviations: Hgb—haemoglobin concentration, PCV--Packed cell volume, MCV-Mean cell volume, MCH—Mean cell haemoglobin, MCHC-Mean cell haemoglobin concentration, WBC-White blood cells*10^9^/l, Plts-Platelets*10^9^/l.

The mean haemoglobin concentration of the male cases was 8.11 ± 1.53 g/dl, packed cell volume 25.04 ± 4.90%, mean cell volume 81.71 ± 6.34 fl, mean cell haemoglobin 26.54 ± 2.46 pg, mean cell haemoglobin concentration 32.45 ± 1.00 g/dl, and male controls mean haemoglobin concentration was13.83 ± 1.32 g/dl, packed cell volume 43.07 ± 3.95%, mean cell volume 86.90 ± 4.69 fl, mean cell haemoglobin 28.25 ± 1.34 pg and mean cell haemoglobin concentration 32.06 ± 0.90 g/dl. For the female cases the mean haemoglobin concentration was 7.78 ± 1.42 g/dl, packed cell volume 23.95 ± 4.49% , mean cell volume 81.36 ± 9.01 fl, mean cell haemoglobin 26.47 ± 3.71 pg , mean cell haemoglobin concentration 32.54 ± 1.08 g/dl. For female controls mean haemoglobin concentration was11.96 ± 3.10 g/dl, packed cell volume 36.59 ± 3.30%, mean cell volume 84.63 ± 8.82 fl, mean cell haemoglobin 27.20 ± 1.92 pg, mean cell haemoglobin concentration 31.59 ± 0.93 g/dl (Table 2).

Majority of the cases 62 of 103 (60.2%) had packed cell volume between 20-30%, followed by 29 of 103 (28.2%) who had values less than 20% and only 12 of 103 (11.7%) had values greater than 30%. Almost all the controls 97 of 98 (99%) had packed cell volume greater than 30%. While only 1 of 98 (1%) had a value less than 30%.

For the cases, the overall mean white blood cell counts was 10.27 ± 3.94 *10^3^/μl and platelet counts of 412.71 ± 145.09*10^3^/μl. While the overall mean white blood cell count for the controls was 5.67 ± 1.59*10^3^/μl and platelet counts of 222.82 ± 57.62*10^3^/μl. The mean white blood cell for males cases was 10.82 ± 4.95*10^3^/μl and platelet counts 408.40 ± 133.42*10^3^/μl and the mean white blood cell count for females cases was 9.83 ± 2.86 *10^3^/μl and platelet counts of 416.12 ± 155.09*10^3^/μl. The mean white blood cell count for males controls was 5.75 ± 1.63*10^3^/μl, and platelet counts of 239.00 ± 62.25 and the mean white blood cell count for females controls was 5.63 ± 1.59; and platelet counts of 222.15 ± 58.03 (Table 2).

Most of the cases 71 of 103 (68.9%) had white blood cell count less than 12,000/μl but a substantial number, 32 of 103 (31.1%) had white blood cell count more than 12,000/μl. Only 1 of 98 controls (1%) had white blood cell count more than 12,000/μl. Cross tabulating white blood cell count with frequency of bone pain showed no statistically significant relationship p value = 0.322.

Almost half of the cases 43 of 103 (41.8%) had platelet count greater than 450,000/μl, while 60 of 103 (58.3%) had platelet count less than 450,000/μl. None of the controls had platelet count greater than 450,000/μl. A statistically significant relationship was found between platelet counts and packed cell volume of the subjects p = 0.001 (Table 3).

**Table 3:** Bivariate analysis of degree of anaemia with platelet counts in cases

	**High Platelets > 450,000/μl**	**Normal Platelets < 450,000/μl**	**Total**	**P value**
PCV				
Low < 20%	17	12	29	0.00
Moderate 20-30%	25	37	62	
Normal >30%	1	11	12	
Total	43	60	103	

Abbreviation. PCV-Packed cell volume.

## Discussion

This study highlights haematologic reference ranges of homozygous sickle cell patients compared with normal controls with haemoglobin phenotypes AA. The red cell indices were generally lower in sickle cell patients than controls with haemoglobin phenotype AA while the white blood cells and platelet counts were higher than control values. These results were expected considering the degree of chronic haemolysis, higher risk of infections and chronic pain in sickle cell patients. Most patients have adapted to low red cell indices; there is therefore no clinical benefit to treat anaemia with blood transfusion. On the contrary, raising the packed cell volume to over 30% could increase blood viscosity, which increases with high packed cell volume [22].

Pain may be responsible for the leucocytosis seen in sickle cell anaemia. The overall mean white blood cells count of 10.27 ± 3.94*10^3^/μl amongst homozygous sickle cell disease patients doubles value obtained in HbAA controls 5.67 ± 1.59*10^3^/μl. Due to re-distribution of the white cells between the marginal and circulating pools, pain, nausea and vomiting and anxiety have been reported to cause leucocytosis in the absence of infection.[23] Although, a statistically significant relationship could not be established in this study between white blood cell count and frequency of pain. Undoubtedly, leucocytosis is associated with poor prognosis, [10-17] while reducing neutrophil count is associated with good prognosis. The benefit of hydroxyurea therapy in sickle cell anaemia follows a fall in neutrophil count, even in patients who have no increase in haemoglobin F [24]

Secondly, and more importantly, leucocytosis in sickle cell disease patients may due to auto splenectomy resulting from recurrent splenic vessels occlusion, which make patients more vulnerable to overwhelming infections particularly, encapsulated organisms like *Streptococcus pneumonia* and *Haemophilus influenzae*.[25]

Despite the controls recruitment being un-intentionally skewed towards female gender who constituted 70% of the volunteers, the overall mean of haemoglobin concentration and packed cell volume of controls 13.83 ± 1.32 g/dl and 43.07 ± 3.95% respectively almost doubles that of cases 7.93 ± 1.47 g/dl and 24.44 ± 4.68% respectively. The rate of chronic haemolysis associated with sickle cell anaemia patients could account for these lower values. There is also a blunted response to erythropoietin secretion in sickle cell anaemia; the rate of increase is not proportional to the degree of anaemia [26]. This may be due to right—shifted haemoglobin dissociation curve seen in sickle cell disease [27]. Similarly lower values were obtained by Omoti in Benin city, Nigeria [28] amongst homozygous sickle cell disease patients in steady state.

The mean cell volume, mean cell haemoglobin, and mean cell haemoglobin concentration are all reduced in anaemia of chronic disease. Expectedly, the mean haemoglobin concentration, packed cell volume, mean cell volume, and mean cell haemoglobin of the controls were higher than cases and males higher than females in both cases and controls. The effects of anaemia of chronic disease, infections and haemolysis could account for lower values seen in cases compared to controls.

Also, confounding factors like cigarette smoking and alcohol intake were ruled out in the cases because insignificant number of them gave history of cigarette smoking and alcohol intake. Increased erythropoesis due to androgens in males, and iron loss or blood loss in females during menstruation may be responsible for higher levels of the red cell indices in males. Reference ranges for erythropoietin are however, not different between the sex. [29] A negative feedback effect on erythropoietin production in males resulting in lower erythropoietin levels would have been expected because of the androgen effect. This indicates that females have better tissue oxygenation for a given haemoglobin level and more efficient tissue red cell delivery.

The overall mean platelet counts for the cases were 412.71 ± 145.09*10^3^/μl and for the controls 222.82 ± 57.62*10^3^/μl which is almost 100% higher in cases compared to controls. A negative feedback effect on erythropoietin production in subjects as a result of the anaemia could be responsible for the thrombocytosis. Erythropoietin has a structural homology with thrombopoetin, although the latter is considerably larger than the former but roughly half of thrombopoetin has identity with or similarity to erythropoietin at the N-terminal region. [30] It is therefore, well recognized that thrombocytosis is associated with anaemia of chronic disease and several types of anaemia.

Reduced or absent splenic sequestration of platelets as a result of hyposplenism in sickle cell disease also contribute significantly to higher mean platelet counts in sickle cell disease compared with controls. [31] This study provides haematologic reference ranges for homozygous sickle cell disease patients compared with normal controls in Lagos, Nigeria. It is our hope, physicians involved in managing sickle cell anaemia patients would become more informed and make use of the findings in this study in their practice.

## Conclusion

Homozygous sickle cell disease patients have lower values of Haemoglobin concentration, packed cell volume, red cell indices, but higher values of white cell count and platelets compared to haemoglobin phenotype AA controls.

## Competing interests

The authors declare that they have no competing interests.

## Authors’ contribution

A.A.A Conceptualized and designed the study. Did the data entry and analysis. D.A.O Drafted the manuscript and revised it critically for important intellectual content. A.A Made substantial contributions to conception and design of the manuscript and revised before final submission. O.O Was involved in the drafting of the manuscript and revised it critically for important intellectual content. A.O - Carried out the full blood count analysis. Dr. A.P - Revised the manuscript critically for important intellectual content and gave final approval of the version to be published. All authors read and approved the final manuscript.

## Limitations of the study

1. All sickle cell cases were diagnosed by only haemoglobin electrophoresis using alkaline buffer. There is no facility to confirm the diagnosis in the centre, some of them might have thalassaemia trait e.g. SBthal which could impact on RBC count, MCV, and haemoglobin concentration.

2. Female patients or controls were not screened for iron deficiency. Presence of iron deficiency in either patients or controls could impact on observed results.

3. Lower haemoglobin in the females may be due to menstrual blood loss.

4. Patients were also not screened for hepatitis B and C infections, known complications of blood transfusion which could induce cytopenias in them.

5. Reliability on background information provided by patients.

6. Lack of case–control matching.
